# Pulmonary thromboendarterectomy at leading high-volume centers: International perspectives

**DOI:** 10.1016/j.jhlto.2026.100550

**Published:** 2026-03-28

**Authors:** Justin Fong, Christine Choi, Natalia Kuzmina, Jacques Thes, Alexander Huang, Antonio Rubino, Sylvain Diop, Dalia Banks

**Affiliations:** a Department of Anaesthesia and Intensive Care Medicine, Royal Papworth Hospital, Cambridge, UK; b Sulpizio Cardiovascular Centre, Department of Anesthesiology, University of California San Diego, La Jolla, California; c Department of Anaesthesia, Toronto General Hospital, University of Toronto, Toronto, Ontario, Canada; d Department of Anesthesiology, Marie Lannelongue Hospital and Paris Saint Joseph Hospital, Paris, Le Plessis-Robinson, France

**Keywords:** chronic thromboembolic pulmonary hypertension, pulmonary embolism, pulmonary thromboendarterectomy, pulmonary hypertension, deep hypothermic cardiac arrest

## Abstract

**Background:**

Chronic thromboembolic pulmonary hypertension (CTEPH) is a potentially curable pulmonary hypertension, caused by persistent obstruction of the pulmonary arteries due to organized thromboembolic material. Pulmonary thromboendarterectomy (PTE) remains the gold standard treatment for eligible patients, offering significant symptomatic relief, improved hemodynamics, and long-term survival. As a complex surgical procedure requiring cardiopulmonary bypass and deep hypothermic circulatory arrest, management of PTE demands a highly specialized, multidisciplinary approach.

**Methods:**

This review represents a collaborative effort from four leading international centers with extensive experience in CTEPH management. We provide a practical overview of perioperative strategies including patient selection, surgical technique, anesthetic management, cardiopulmonary bypass considerations, and postoperative intensive care management. Institutional variations in practice were also compared.

**Results:**

Shared principles across centers included careful hemodynamic optimization, advanced perioperative monitoring, and structured postoperative critical care. Areas of institutional variation included anticoagulation approaches, monitoring practices, neuroprotection techniques, blood conservation strategies, postoperative ventilation, and hemodynamic management. These differences reflected local protocols developed in the setting of limited comparative evidence.

**Conclusions:**

PTE remains the definitive treatment for operable CTEPH at experienced centers. Successful outcomes rely not only on surgical expertise but also on coordinated anesthetic and critical care management. This review summarizes current international practice, highlights areas of consensus and variability, and identifies priorities for future multicenter evaluation and quality improvement initiatives.

## Background

Pulmonary hypertension (PH) is a complex clinical syndrome defined by a mean pulmonary artery (PA) pressure greater than 20 mm Hg at rest, as measured by right heart catheterization. It encompasses a spectrum of diseases with diverse etiologies, pathophysiology, and treatment strategies. The World Health Organization classifies PH into 5 major groups depending on the etiology (Table 1) with chronic thromboembolic pulmonary hypertension (CTEPH) being in group 4.

**Table 1:** Classification of Pulmonary Hypertension

Group	Classification
Group 1	Pulmonary arterial hypertension
Group 2	PH due to left heart disease
Group 3	PH due to lung disease/hypoxia
Group 4	Chronic thromboembolic PH
Group 5	PH due to unclear or multifactorial mechanism

Abbreviations: PH, pulmonary hypertension.

Adapted from Humbert et al, 2022 European Society of Cardiology / European Respiratory Society Guidelines for the diagnosis and treatment of pulmonary hypertension.^1^

Each group has distinct underlying causes and management strategies. While most forms of PH are medically managed, CTEPH is unique in that it is potentially curable through surgical intervention. CTEPH results from persistent obstruction of the pulmonary arteries following an acute pulmonary embolism. This obstruction is not only due to organized thromboembolic material but also involves secondary vascular remodeling, including intimal hyperplasia and medial hypertrophy of the small pulmonary arteries and arterioles.

Pulmonary thromboendarterectomy (PTE), also referred to as pulmonary endarterectomy in the literature, remains the treatment of choice for eligible patients and is associated with excellent outcomes. Perioperative mortality has significantly improved over the past 2 decades and is now below 2% at experienced centers. However, a substantial proportion of patients are deemed inoperable due to anatomical or clinical factors. Hence, an individualized management by a multidisciplinary team is essential. Three key treatment modalities include pulmonary endarterectomy, balloon pulmonary angioplasty, and targeted medical therapy.

## Preoperative management

Successful PTE surgery begins with a robust process for patient identification and surgical candidate selection. CTEPH is a progressive disease that, if left untreated, ultimately leads to right ventricular failure, underscoring the critical importance of early diagnosis and timely intervention. Due to the subtle and nonspecific symptoms, CTEPH is likely underdiagnosed in the broader community. This highlights the need for increased awareness and systematic evaluation, particularly from primary care clinicians who are often the first point of contact.

**Figure 1:**
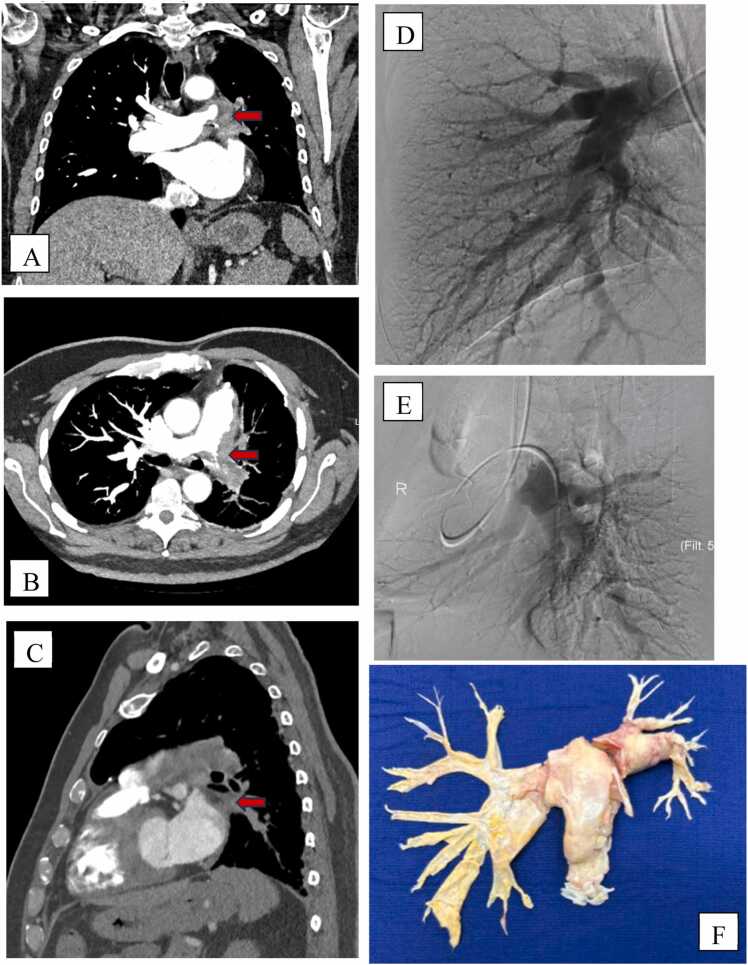
CT pulmonary angiogram of chest: (A) coronal, (B) axial, and (C) sagittal views. Pulmonary angiogram of (D) right lung and (E) left lung. (F) Excised thrombotic clot material.

The re--ed screening test is a Ventilation/Perfusion (V/Q) scan, while pulmonary angiography is the gold standard for diagnosis. Computed tomography pulmonary angiography or magnetic resonance imaging may be used as additional diagnostic tools (Figure 1).^2^

The decision to offer a PTE is complex and requires input from a multidisciplinary team, ideally consisting of a PTE surgeon, CTEPH/PH specialist, balloon pulmonary angioplasty interventionalists, and a thoracic radiologist. Major considerations influencing surgical candidacy include the proximal location of the diseased segments, the likelihood of a significant reduction in pulmonary vascular resistance (PVR) following surgery, and the absence of comorbidities that would prohibit cardiac surgery or engagement in the recovery process.^3^

Preoperative right heart catheterization is useful for identifying the severity of PH, while transthoracic echocardiography is essential in the assessment of biventricular failure, and identification of interatrial septal defect or valvular pathology that may warrant concomitant repair. Coronary angiography is re--ed in patients with risk factors for coronary artery disease, including advanced age and relevant comorbidities.

Before surgical intervention, patients should undergo medical optimization of comorbidities and a comprehensive preanesthetic evaluation. Sequelae of right heart failure, such as hepatic and renal dysfunction, should be identified as they may significantly impact postoperative recovery. Irrespective of whether a specific hematological diagnosis is made, patients are presumed to be in a hypercoagulable state and should receive anticoagulation from the time of diagnosis up until the time of surgery, with appropriate bridging therapy.^4^

Preoperative anticoagulation strategies are broadly aligned across participating centers, with the objective of maintaining continuous therapeutic anticoagulation until the time of surgery. Most centers transition patients from oral anticoagulants to IV unfractionated heparin or low-molecular-weight heparin in the days preceding surgery, allowing rapid titration and perioperative control. In patients with antiphospholipid syndrome or other high-risk thrombophilic conditions, management is individualized in coordination with hematology specialists, typically maintaining uninterrupted parenteral anticoagulation before surgery. Specific bridging protocols and monitoring strategies (e.g., anti-Xa–guided dosing) may vary between institutions, reflecting local practice patterns rather than evidence demonstrating superiority of one approach.

Although less commonly today, the presence of an inferior vena cava filter should also be noted, as this will affect venous cannulation if venous-venous (VV) or peripheral venous arterial (VA) extracorporeal membrane oxygenation (ECMO) is being considered. Disease-specific medications, such as pulmonary vasodilators, can be continued into the perioperative period but can be tapered or discontinued postoperatively if the surgical outcome is favorable and pulmonary hemodynamics improve. Preoperative fluid balance optimization is critical as even small changes in right atrial pressure can significantly impact right ventricle (RV) function and tolerance of anesthesia. Intraoperative management must be carefully planned to prevent RV failure and sudden hemodynamic collapse, especially given the typically borderline RV function in this patient population.

Given that PTE is the definitive treatment for CTEPH, the degree of physiologic optimization before surgery is inherently limited. This underscores the critical importance of meticulous anesthetic management.

## Intraoperative management

### Induction of anesthesia and prebypass period

Upon entering the operating room, standard anesthesia monitors such as electrocardiogram and pulse oximetry are applied. Before induction of anesthesia, large-bore peripheral intravenous (IV) access for potential vasoactive medication administration and an arterial line for close hemodynamic monitoring should be established. Benzodiazepines or opioids for anxiolysis should be used with caution, as respiratory depression can increase PVR, potentially leading to RV failure and hemodynamic collapse. Induction agents are selected according to institutional preference and patient physiology. Propofol or etomidate are commonly used, and some centers incorporate low-dose ketamine within a balanced technique when clinically appropriate. Emphasis is placed on careful titration, avoidance of systemic hypotension, prevention of acute increases in PVR, and readiness to initiate vasoactive support. Hemodynamic support is guided by real-time assessment of right ventricular function and systemic perfusion goals rather than by the specific induction agent selected. During induction, careful control of ventilation is prioritized to avoid hypoxia and hypercarbia, which may increase PVR and precipitate right ventricular instability. Neuromuscular blockade facilitates controlled airway management; however, the specific sequence and timing of intubation are individualized according to patient physiology and institutional practice. Transesophageal echocardiography is typically introduced early during the induction period and provides a valuable real-time assessment of right ventricular function and hemodynamic response. Hemodynamic support during induction and maintenance is guided by right ventricular function and systemic perfusion goals. Short-acting vasoconstrictors (e.g., metaraminol or phenylephrine) are frequently used during induction to promptly treat systemic hypotension, while vasopressor infusions such as norepinephrine or vasopressin may be initiated when sustained support is required. Inotropes such as dopamine or epinephrine are introduced in cases of impaired right ventricular contractility as assessed by transesophageal echocardiography. Intubation with a large-size endotracheal tube is encouraged to facilitate bronchoscopy in case of pulmonary hemorrhage or pulmonary edema during the postbypass period.^5^ A transesophageal echocardiogram (TEE) probe is inserted after intubation to guide pulmonary artery catheter (PAC) insertion, detect right atrial or proximal PA thrombus, and for real-time cardiac function assessment and hemodynamic management.^6^ Central venous access and PA catheterization can be performed preinduction or postinduction depending on institutional protocol. The right internal jugular vein is typically chosen for ease of access. While preinduction PAC placement can allow close management of filling pressures, PVR, and RV output during induction of anesthesia, some patients may not tolerate being supine due to anxiety and dyspnea which may lead to worsening PVR through hypoventilation and hypercarbia.^7^ Additionally, the PAC can be technically difficult to float due to the presence of severe tricuspid regurgitation and a dilated PA.^8^ A femoral arterial line is placed postinduction as the radial arterial line often becomes unreliable over the course of the operation with peripheral vasoconstriction from prolonged periods of hypothermic cardiopulmonary bypass (CPB) and deep hypothermic circulatory arrest (DHCA).^9^ Electroencephalography and cerebral oximetry monitors are applied for cerebral monitoring, and the head is wrapped with a cooling device to ensure adequate and even cooling of the brain for DHCA.^5^

The pre-CPB period is relatively short. A median sternotomy is performed, and the pericardium is opened for surgical exposure of the heart. IV heparin is given, and the aorta and vena cavae are cannulated. Institutional heparin protocols vary with most centers administering 400 IU/kg of heparin.^10^, ^11^, ^12^ Due to the history of chronic pulmonary embolism and frequent prior exposure to anticoagulation, heparin resistance is not uncommon.^13^ Routine preoperative screening for antithrombin III deficiency is not systematically performed across participating centers. However, in cases of inadequate activated clotting time response despite appropriate heparin dosing, supplementation with antithrombin III concentrate or fresh frozen plasma may be considered. This approach is guided by intraoperative anticoagulation monitoring rather than by predefined dosing protocols. Before initiation of CPB, the presence of a patent foramen ovale (PFO) can be assessed using transesophageal echocardiography and agitated saline contrast. The decision to close a PFO during PTE varies between institutions and is typically individualized based on shunt magnitude, right ventricular pressure profile, and anticipated postoperative hemodynamics. While some centers routinely close identified PFOs, others adopt a selective approach, intervening primarily when shunting is hemodynamically significant. Once an adequate activated clotting time is achieved, CBP is initiated, and the patient's core body temperature is cooled to below 20°C. During the prebypass period, cardiac output/index and PVR are continuously monitored.

### Initial CPB period—Cooling, circulatory arrest, and thromboendarterectomy

The CPB pump is primed following standard institutional protocols. Methylprednisolone serves as a cell membrane stabilizer and anti-inflammatory agent. Routes of administration and doses vary between centers, depending on established practice. Some centers avoid steroid use altogether, citing a lack of strong evidence showing clinical benefit.^14^ Tranexamic acid (TXA) is used by most, but not all, centers to reduce bleeding during PTE. Institutional variation in its routine use reflects differences in historical protocol development and interpretation of thrombotic risk in the CTEPH population. While antifibrinolytic therapy is well established in cardiac surgery more broadly, PTE-specific comparative data remain limited. Consideration of proconvulsant potential and thrombotic risk is particularly relevant in the context of DHCA.^15^ Dosing strategies vary among institutions and may be adjusted according to patient factors such as renal function and surgical complexity.^16^ Some centers have observed reductions in blood product utilization following implementation of routine TXA protocols, without apparent increases in thromboembolic complications. However, no multicenter data currently link the TXA strategy to differences in re-exploration or long-term outcomes in PTE.

The optimal hematocrit level during DHA and CPB for adequate cerebral perfusion and oxygen delivery is unknown.^17^, ^18^, ^19^, ^20^ As part of a blood conservation strategy, sequestration of 500 to 1,000 ml of autologous blood may be considered before CPB to achieve a hematocrit of 22% to 28%, thereby reducing the need for transfusion.^12^, ^21^ Intraoperative cell salvage is routinely employed across participating centers as part of standard cardiac surgical blood conservation practice. Point-of-care viscoelastic coagulation testing, such as rotational thromboelastometry, has been shown to reduce perioperative blood loss and blood product utilization in high-risk cardiothoracic surgery, supporting its role in structured transfusion algorithms.^22^ Alpha stat management is typically used for acid-base management on CPB. Cooling is performed to achieve a core temperature of 20°C. Once tympanic membrane temperature reaches ≤18°C, bladder or rectal temperature is ≤20°C, DHCA can be performed. Propofol can be given at a dose of 2.5 mg/kg immediately before DHCA to achieve complete cerebral isoelectricity.

**Figure 2:**
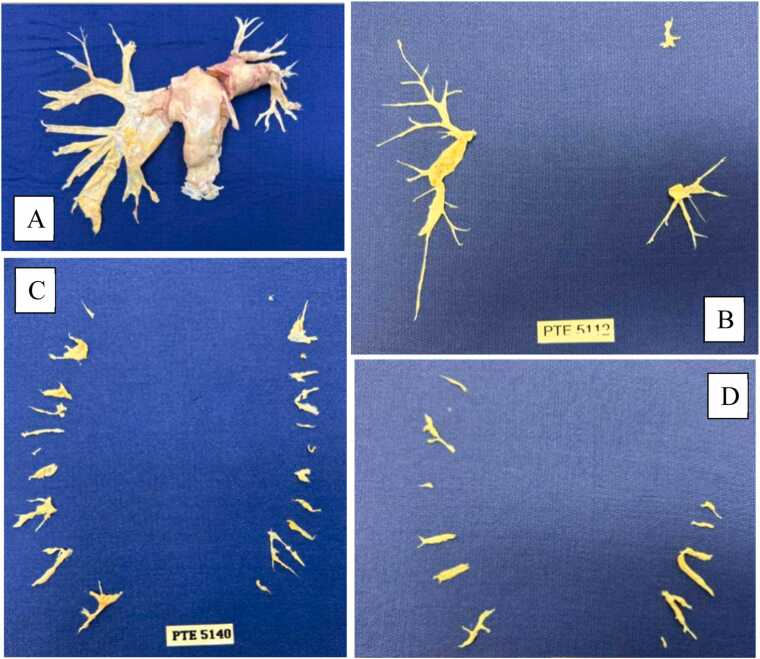
Excised thrombotic material: (A) level I disease on right and level IC disease on left, (B) bilateral level II disease, (C) bilateral level III disease, and (D) bilateral level IV disease. IV, intravenous; PTE, pulmonary thromboendarterectomy.

Thrombotic clots and fibrotic material adherent to the intima are surgically excised during DHCA. The duration of DHCA should not exceed 20 minutes at a time to limit neurological consequences.^5^, ^9^ This approach is consistent with expert consensus and published CPB temperature management guidelines, which support limiting individual periods of DHCA to minimize neurological risk in the absence of definitive comparative trial data. If additional time is required, CPB is resumed for 10 minutes before initiating another DHCA period. Once satisfactory removal of the thrombotic material is achieved, rewarming is initiated. The anatomic distribution of the excised thrombotic material will determine the CTEPH disease classification (Table 2). Higher level, more proximal disease is more surgically accessible, while more distal lesions increase procedural complexity and risk of complication (Figure 2).

**Table 2:** University of California San Diego Chronic Thromboembolic Disease Classification

Classification	Disease location
Level 0	No disease in lung
Level I	Main pulmonary artery
Level IC	Complete occlusion of one main pulmonary artery
Level II	Lobar artery or main descending pulmonary artery
Level III	Segmental artery
Level IV	Subsegmental artery

Based on the previously described anatomic surgical classification of CTEPH disease distribution.^23^

### Rewarming period

Rewarming is a gradual process with a maximum rate of 0.5°C per minute. To prevent thermal injury, the arterio-venous gradient should not exceed 10°C when the arterial temperature is below 30°C, and 5°C when the temperature is above 30°C.^24^ Rewarming must be carefully controlled to avoid endogenous bubble formation secondary to the acute change in blood gas solubility as a result of temperature variation.^24^, ^25^, ^26^ To limit the risk of cerebral injury related to hyperthermia, the temperature of the CPB arterial outlet line is kept below 37°C. Before weaning from CPB, venous inflow, as well as core temperature (vesical or rectal), should both be above 36°C.^24^

Ventricular fibrillation is common during rewarming and often resolves spontaneously or through the administration of antiarrhythmic drugs such as magnesium sulfate or lidocaine. If no spontaneous cardiac rhythm has resumed, an internal defibrillation of 10 J is delivered to restore sinus rhythm. Atrial pacing may be necessary to maintain an adequate cardiac rate and rhythm.^5^ Electrolyte and hematologic disturbances should be corrected if necessary. Hemolysis in the urine may be noted and is not unexpected. The potential need for hemostatic agents is anticipated according to patient baseline hematological abnormalities, clinical assessment, and conventional or point-of-care coagulation parameters. The use of adjunctive osmotic diuretics such as mannitol during CPB varies between institutions and is not routine across all centers. When administered, it is typically guided by intraoperative considerations, including urine output, hemolysis, and renal perfusion status rather than by a standardized protocol.

Ventilation is gradually reintroduced during rewarming, initially with minimal tidal volumes, before full lung expansion before separation from CPB. Tidal volumes are typically calculated using ideal body weight (IBW) and lung-protective principles (approximately 6 ml/kg IBW); however, some centers base calculations on actual or adjusted body weight, which may result in higher delivered tidal volumes. Recruitment maneuvers are performed before CPB discontinuation to minimize atelectasis. Moderate levels of positive end-expiratory pressure (PEEP) are applied to reduce reperfusion lung injury while avoiding excessive intrathoracic pressure that could compromise right ventricular function.

### Weaning from CPB and post-CPB

In preparation to wean off CPB, mean arterial pressure (MAP) is maintained above 65 mm Hg with the use of vasoactive medications as needed. The heart rate is kept above 80 beats per minute with atrial pacing, if necessary, to avoid right ventricular overdistension and to optimize cardiac output. Right and left ventricular function is assessed by TEE. Cardiac output measurements and mixed venous O_2_ saturation monitoring by PAC are resumed. Mechanical ventilation parameters are optimized per institutional norm. Inotropic support and pulmonary vasodilators, such as inhaled nitric oxide (iNO) and prostacyclin, are initiated if required. Pulmonary vasodilator therapy is not routinely employed but may be introduced selectively in cases of difficulty separating from CPB due to persistent right ventricular dysfunction or elevated PVR. iNO is most commonly used in this context. Institutional thresholds for pharmacologic escalation vs early initiation of VA-ECMO vary, and in some centers, a low threshold for mechanical support is favored over prolonged vasodilator therapy. CPB flow is gradually reduced and then discontinued once hemodynamic stability has been achieved. After separation from CPB, unfractionated heparin is reversed with protamine. The patient's blood contained in the CPB venous reservoir can be slowly reinfused before decannulation or can be processed through a cell-salvaging system based on filling pressures and hemodynamic parameters.

Coagulopathy, though uncommon, can be corrected in cases of abnormal bleeding based on laboratory values and point-of-care coagulation testing. Preload and filling pressures are continuously assessed by TEE and PAC, with an objective to maintain CO adequate for tissue perfusion, while minimizing the risk and severity of reperfusion pulmonary edema.

### Perioperative complications

Several complications may occur during rewarming and separation from CPB. The most significant are airway hemorrhage, reperfusion pulmonary edema, residual PH, and right ventricular dysfunction leading to a low cardiac output syndrome.^5^

Airway hemorrhage following PTE occurs in approximately 1% to 6.6% of cases and carries significant mortality.^27^, ^28^, ^29^ It varies in severity and often manifests during rewarming, when transpulmonary flow is gradually resumed.^4^ For this reason, close communication between the anesthesia, surgical, and perfusion teams is essential during this critical period. The most common etiology of bleeding is injury to the PA during endarterectomy, though systemic hemorrhage from bronchial arteries can occur.^4^ Clinical management focuses on reversal of anticoagulation and coagulopathy, isolation of bleeding lung segments, and application of higher PEEP. In case of minor bleeding that does not compromise oxygenation, separation from CPB, reversal of heparin, and administration of topical hemostatic agents (TXA/epinephrine/vasopressin) will promote hemostasis.^4^, ^5^ In case of severe bleeding, full flow CPB must be re-established to reduce pulmonary perfusion.^5^ Bleeding may still persist due to the ongoing bronchial blood flow. If bleeding is unilateral, isolation of the source by double-lumen tube, bronchial blocker, or segmental occlusion via balloon inflation may allow separation from CPB and heparin reversal.^4^, ^5^ When CPB separation is difficult due to compromised oxygenation or RV failure, VV- or VA-ECMO is useful as a bridge to hemostasis and recovery.^4^, ^5^ In rare cases of refractory bleeding, embolization of affected segments or pulmonary resection might be necessary.

Residual PH and RV dysfunction are often linked to persistent distal vasculopathy with incomplete surgical clearance.^4^ It can be exacerbated by hypoxemia and hypercapnia related to reperfusion edema or airway bleeding.^5^ Inotropic support and inhaled pulmonary vasodilators can help to overcome RV dysfunction in some cases, but in the most severe situations, VA-ECMO implementation may be necessary.^5^ Cannulation can be central or peripheral.^30^

Reperfusion pulmonary edema occurs in up to 40% of cases following PTE.^5^ It results from sudden restoration of blood flow in the previously occluded large pulmonary vascular territories, leading to impaired oxygenation from ventilation perfusion mismatch, and a decrease in diffusion capacity. Treatment is generally supportive and follows principles of acute respiratory distress syndrome management. Severe hypoxemia may require inhaled NO and temporary VV-ECMO. Cardiac output is maintained at a subnormal level to limit pulmonary flow, and positive fluid balance is avoided.

### Postoperative period

Postoperative management is overseen by a multidisciplinary team involving intensivists (often with an anesthesia background), surgeons, and pulmonologists, depending on institutional structure and expertise. This is a critical period in which awareness of key principles of clinical management is essential to ensure optimal recovery.

Transport to the intensive care unit (ICU) is typically carried out with sedation and either manual or portable ventilator support, ensuring lung protection and effective CO₂ control. Chest tube output should be closely monitored throughout transport and in the ICU. Once in the ICU, sedation is continued most frequently with propofol and opioids as adjunct therapy. Benzodiazepines are avoided to facilitate weaning and preparation for extubation.^31^ Lung-protective mechanical ventilation, with individually titrated PEEP and an FiO_2_ as low as clinically appropriate, is preferred. As long as plateau and driving ventilatory pressures remain relatively low, tidal volume (Vt) up to 10 ml/kg IBW does not seem to adversely affect outcomes.^32^, ^33^ Ventilation is balanced to avoid hypercarbia, barotrauma, and lung injury. The timing of extubation varies between centers. For patients requiring a period of monitoring and optimization before extubation, diuresis is initiated to target a negative fluid balance, usually in the first 12 to 36 hours postoperatively.^34^ Early extubation is pursued in most patients once hemodynamic stability and adequate gas exchange are achieved. However, the early postoperative period (first 24-48 hours) carries a recognized risk of reperfusion pulmonary edema, particularly in patients with severe preoperative PH, which may necessitate continued ventilatory support. As such, while ICU stays are often short, formal “fast-track” pathways are less focused on immediate extubation and more closely linked to early mobilization, chest drain management, and timely transfer to rehabilitation when appropriate. There are no fast-track protocols described in the PTE population, and the benefits of immediate extubation are unclear. Gas exchange criteria for extubation are similar between individual centers, with common thresholds of FiO2 35% to 40%, PEEP 4 to 6 cm H_2_O, and pO_2_ > 75 mm Hg.

Hemodynamic management is another cornerstone of postoperative care. Initially, the femoral artery is the preferred site for MAP monitoring in most centers due to greater reliability in the early postcirculatory arrest and postbypass period compared to radial artery measurements.^9^ Cardiac output and mixed venous saturation are measured continuously or intermittently at the preference of each center. Hemodynamic goals focus on ensuring adequate RV perfusion pressure and include maintaining a MAP ≥ 65 mm Hg and aggressive treatment of arrhythmias.^35^ Cardiac output management is aimed at the maintenance of sufficient end-organ perfusion while preventing hyperdynamic pulmonary circulation. This may involve fluid administration or diuresis. Maintenance fluids are not routinely administered; instead, a restrictive, goal-directed approach is adopted.^4^ Balanced crystalloids are most commonly used for intravascular volume support, while albumin may be incorporated in CPB priming or selectively in specific clinical contexts. At present, there are no PTE-specific data demonstrating the superiority of one fluid strategy over another. This may be guided by cardiac output measurements via the PAC and monitoring of biochemical parameters such as lactate and renal function. Diuresis to achieve a negative fluid balance is routinely pursued in the early postoperative period, most commonly using loop diuretics initiated in the ICU.^4^, ^34^, ^36^ The timing and intensity of diuretic therapy may vary according to hemodynamic stability, reperfusion lung injury, and evidence of hemolysis. Some centers also employ mannitol for osmotic diuresis.^4^ Pulmonary vasodilators are not used routinely to avoid excessive pulmonary circulation.^33^ If a reduction in RV afterload is needed, iNO is the agent of choice.

Therapeutic anticoagulation starts in most centers within the first 24 hours postoperatively.^34^ Initial anticoagulant choice depends on the individual center’s experience, but heparin and low molecular weight heparin are most frequently used. Chest tube output is carefully monitored after the start of therapeutic anticoagulation. In most uncomplicated cases, chest tubes are removed within the first 48 hours postoperatively.^4^ ICU length of stay is on average 2 to 4 days, dependent on the center.^4^, ^34^, ^36^

### Postoperative complications

Hemodynamic instability is common in the early postoperative period and should be managed based on the underlying etiology. PAC monitoring and transthoracic echocardiography are frequently used to assist with diagnosis and treatment. Management focuses on the maintenance of RV preload and perfusion pressure to avoid RV failure. Tachyarrhythmias are treated aggressively, while bradycardia is managed with temporary pacing. Vasopressors such as norepinephrine or vasopressin are commonly used to maintain right ventricular perfusion pressure and systemic stability. Low-dose vasopressin may offer theoretical advantages in this setting by increasing systemic vascular resistance without directly stimulating β-adrenergic receptors and with minimal pulmonary vasoconstrictive effect at physiologic doses. However, potential risks such as excessive vasoconstriction or reflex bradycardia must be considered, and no PTE-specific comparative data currently demonstrate superiority over catecholamine-based strategies. Catecholamines, including dopamine or dobutamine, may be employed according to the predominant hemodynamic profile and right ventricular performance. The choice of agent varies between institutions and reflects local experience and protocol development rather than evidence demonstrating the superiority of one vasoactive strategy over another in the PTE population.^4^ In cases of low cardiac output, increasing serum lactate levels and signs of hypoperfusion, catecholamines, such as dobutamine or epinephrine, are considered. If residual PH contributes to RV dysfunction, RV afterload reduction is initiated using iNO or iloprost.^23^, ^34^ VA-ECMO or VV-ECMO with a dual-stage cannula can be considered in severe RV insufficiency. Ongoing hemorrhage is treated with appropriate transfusion. Hypoxemia in the early postoperative period is often multifactorial. Important causes include undiagnosed intracardiac shunts, left-sided heart failure, pulmonary hemorrhage, and reperfusion pulmonary edema.

Reperfusion pulmonary edema is not uncommon, presenting within the first 24 to 48 hours postoperatively.^14^, ^23^, ^33^, ^37^, ^38^ Management consists of aggressive diuresis, protective lung ventilation, and positional therapy to reduce perfusion of the affected lung.^34^ Catecholamines are avoided to prevent hyperdynamic cardiac output if there are no signs of systemic hypoperfusion.^33^ iNO facilitates oxygenation without augmenting blood flow and can be useful. In refractory cases, VV-ECMO should be considered to maintain oxygenation.^39^

Management of pulmonary hemorrhage in the ICU follows the same diagnostic, therapeutic, and interventional measures as in the operating room, involving a multidisciplinary approach depending on the nature of bleeding.^34^, ^40^ High chest tube output warrants investigation. Coagulopathy, if present, should be promptly diagnosed using both conventional and point-of-care viscoelastic coagulation testing and treated accordingly. The decision to continue or stop anticoagulation in case of increased chest tube output is made jointly with the surgical team.

Acute kidney injury can affect as many as 49% of patients undergoing PTE. Acute kidney injury stage III affects 1% to 10% of patients in centers presented.^41^ Management is generally supportive, by maintaining kidney perfusion, optimizing volume status, while avoiding excessive pulmonary blood flow and edema. Continuous renal replacement therapy is mostly employed to reach a negative net balance in oliguric patients. Anticoagulation for continuous renal replacement therapy is discussed with the surgical team, and citrate is used when heparin is contraindicated.

## Conclusion

Although the fundamental anesthetic and critical care principles underpinning PTE have remained stable over the past 2 decades at experienced centers, several aspects of perioperative management have evolved. These include broader adoption of advanced neuromonitoring during DHCA, routine use of transesophageal echocardiography to guide intraoperative decision-making, expanded availability of extracorporeal life support for refractory right ventricular failure or severe reperfusion lung injury, and increasingly individualized postoperative ventilation and fluid management strategies.

Despite these advances, important practice variation persists among high-volume centers. Such differences largely reflect institutional experience, protocol development, and the absence of robust comparative data rather than disagreement regarding core physiologic principles. Across centers, shared priorities remain consistent: meticulous control of PVR, avoidance of right ventricular overdistension, and early recognition and management of reperfusion injury. At present, no multicenter data demonstrate the superiority of any specific anesthetic or critical care strategy following PTE.

Patients undergoing PTE for CTEPH represent a uniquely high-risk surgical population due to the severity of their underlying pathophysiology and the complexity of intraoperative and postoperative management. Optimal outcomes depend on a multidisciplinary, protocol-driven approach throughout the entire perioperative period, with a significant emphasis on patient selection and preoperative optimization. Surgery is considered curative, with immediate reductions in PVR observed in the operating theater. In the setting of an uncomplicated intraoperative period, these patients respond well to surgical clearance, requiring minimal administration of blood products, inotropes, or vasopressors postoperatively when compared to other high-risk cardiothoracic surgical populations.

Given the potential of life-threatening complications, centralization of PTE to high-volume centers is essential. Concentrated expertise in surgical technique, anesthetic management, and cardiothoracic intensive care—including ready access to ECMO—has been consistently associated with improved outcomes (Table 3 for institutional protocol differences). Future research in perioperative management of PTE should focus on optimization of right ventricular support strategies, refining lung-protective ventilation during and after CPB, and neuroprotection during hypothermic circulatory arrest. Additional investigation into renal protection strategies and structured enhanced recovery pathways may further reduce morbidity and accelerate rehabilitation. Ultimately, multicenter collaboration and prospective registry data will be essential to determine whether specific perioperative strategies translate into meaningful differences in patient-centered outcomes.

**Table 3:** Institutional Differences

	Royal Papworth	UC San Diego	Marie Lannelongue	Toronto General
Central venous line timing	Postinduction	Postinduction	Postinduction	Patient-dependent
Initial dose of heparin (U/kg)	400	400	300	400
Heparin alternative for urgent surgery in the context of HIT	Postpone surgery until antibody negative then heparin Postoperative: argatroban or fondaparinux	Intraoperative: cangrelor with heparin^42^ Postoperative: bivalirudin	Intraoperative: cangrelor with heparin Postoperative: argatroban	Plasmapheresis then heparin Postoperative: argatroban
ACT target	500	450	450	480
Methylprednisolone administration	500 mg IV	30 mg/kg as bypass prime - max 3 g)	Nil	1,000 mg IV
Seizure prophylaxis	Nil	Fosphenytoin 15 mg/kg	Thiopental 1 g at 20°C	Nil
Tranexamic acid	1,000 mg bolus + 500 mg/h	Nil	10 mg/kg bolus + 10 mg/kg/h	30 mg/kg bolus + 15 mg/kg/h
Venesection before CPB	Yes (HCT 22%-26%)	No	Yes (HCT 24%-28%)	No
Hemoglobin threshold before coming off CPG (g/liter)	>100	>80	>80	>80
Routine TEG/ROTEM	Yes	No	Yes	Yes
Protamine dosing	1:1	1:1	0.8:1	0.8:1
Ventilation parameters (Vt of IBW) (ml/kg)	8	10	6	6-8

Abbreviations: ACT, activated clotting time; CPB, cardiopulmonary bypass; HCT, hematocrit; HIT, heparin-induced thrombocytopaenia; IBW, ideal body weight; ROTEM, rotational thromboelastometry; TEG, thromboelastography; Vt, tidal volume.

## CRediT authorship contribution statement

**Justin Fong:** Writing – original draft, Conceptualization, Visualization, Writing – review & editing. **Christine Choi:** Writing – original draft, Writing – review & editing, Data curation. **Natalia Kuzmina:** Writing – original draft (initial draft), Investigation. **Jacques Thes:** Supervision, Writing – review & editing. **Alexander Huang:** Conceptualization, Supervision, Writing – review & editing. **Antonio Rubino:** Conceptualization, Supervision, Writing – review & editing, Project administration. **Sylvain Diop:** Writing – review & editing, Resources. **Dalia Banks:** Conceptualization, Supervision, Writing – review & editing.

## Declaration of Competing Interest

All authors declare that they have no known competing financial interests or personal relationships that could have appeared to influence the work reported in this paper.
